# Supplementary material for: Manipulating risk of infection and appeal to public benefit increase compliance with infection control measures in a hypothetical pandemic scenario
Source: PLoS One. 2022 Nov 22;17(11):e0274024. doi: 10.1371/journal.pone.0274024 (PMC9681092; doi:10.1371/journal.pone.0274024)
Supplement: Attachment — Submitted filename: Review_PONE-D-22-05740.pdf [file pone.0274024.s002.pdf]

Thank you for the opportunity to review the manuscript. The work is very interesting in terms of exploring different motivations for adherence to infection control measures during pandemics. However, it is unclear what recommendations for policy makers can be derived from the study results and to what extent best practices and ethical standards of effective (evidence-based) risk communication have been considered, aiming at empowering citizens in democratic societies to make informed decisions. For instance, evidence suggests that persuasive strategies threaten trustworthiness and credibility of the communicator and of vaccinations (e.g., Blastland et al. 2020; Kachurka et al. 2021; Volpp et al. 2021. Therefore, trust in public health authorities is a key factor in behaviour and compliance. We suggest, that different strategies of communication with the public as well as their chances of success and consequences should at least be discussed.

What is missing from the limitations is that the results are not transferable to other countries, as trust in national public health authorities and past risk communication (especially the COVID-19 risk communication) influence the trust of the population and thus have an impact on compliance with future infection control measures.
Further, there is no discussion in the limitations section on how the under-representation of certain groups (e.g., with low education) can be reduced in the future. There is also the question whether other factors besides education, age, gender that have an influence on compliance with protection measures (e.g., SES, race/ethnicity) have been considered. Can they be identified using the Norwegian Citizen Panel?

Please find below a few comments on some of the sections and phrases.

Abstract

In general, the methodology could be explained a bit more: sample, design, analyses.

It is not clear from the abstract what the main objective is and in how far the results of this study can inform future communication of health authorities. In 1.3.1 you wrote *"The aim of the current study is to test causal relationships between risk of infection and type of motivation on complying with infection control measures, and to examine if these factors interact."* and in line 119 "*Knowledge about the relative importance of self-interested and prosocial motives in different risk scenarios can inform health authorities about communication strategies during different phases of disease outbreak*." Maybe you can add this to your abstract.

Line 17 *„Perceiving the situation as threatening and seeing public benefits to complying may increase the public's motivation to comply."*

It is not clear what the conclusion for political actions could be? Should it be the aim of political communication be to raise fear (even if there is no reason for it or when there is a lot of uncertainty)? We view this statement critically in the light of evidence-based risk communication, which aims at transparent and balanced risk communication to enable informed decisions. If persuasion is the recommendation, it should be made more clear in the abstract.

Introduction

Line 38 "*Although the probability of an event and the expected outcome can be established statistically, perceived risk has been shown to deviate from objective measures of risk (2,3). How threatening a pandemic is perceived to be, may be influenced by how public authorities communicate the risk scenario.*"

The statistical probabilities of occurrence cannot be determined for all events, as also described in the paper by Mousavi & Gigerenzer 2014 that you cite. Especially during the COVID-19 pandemic, a great deal of uncertainty existed at the beginning (and to some extent still exists). Therefore, a key factor influencing perceived risk is how risks and uncertainties are communicated by public authorities. However, evidence-based and user-centered risk communication not only includes transparent and balanced communication of probabilities, but also indicates the uncertainties, and takes into account the needs of different target groups). People better understand risks when, for instance, risk probabilities are communicated numerically instead of verbally (for example, "5 in 100 people will be infected" can be grasped more concretely than "The risk of infection is low or high"), since people tend to interpret verbal probability statements differently. Simple frequencies or per- centages (for example, 5 in 100 or 5%) are more comprehensible than probabilities or 1-in-x formats (for in- stance, 1 in 20). By communicating both numerator and denominator, it can be conveyed whether the risk is big or small. Moreover, when comparing risks, the denominator should be kept the same (for example always 100). Relative risk reductions are unclear ("The intervention reduced the number of infections by 20%"), in- stead, absolute risk reductions are recommended ("The intervention reduced the number of infections from 5 in 100 people without the treatment to 4 in 100 people with the treatment") (e.g., Zipkin et al. 2014, Büchter et al. 2014). The latter illustrate the absolute size of a risk. If there is insufficient information to report numbers, the reason for this should be stated. Visual presentations can be a beneficial supplement or substitute for numerical or verbal information on risks. They can facilitate comprehension, especially for people with low numeracy or reading skills (e.g., McDowell et al. 2021).

Line 60 "*If feeling threatened is essential for compliance, this would justify that health authorities emphasize personal and societal risks when promoting infection control measures.*"

In the light of evidence-based risk communication, the benefits and harms of both perspectives (personal and societal risks) should be communicated if this is in the interest of the addressees.

Line 112 "*How people perceive risk is related to their ability to control their level of exposure and it may thus be difficult to determine the impact of perceived risk on compliance from correlational data alone (25).*"

How people perceive risks also depends on their risk literacy, which in turn is influenced, for example, by their statistical skills and the way in which risks, and uncertainties are communicated (e.g., Cokely et al. 2018). If risks are not correctly understood, risks can be overestimated or underestimated (e.g., Wegwarth et al. 2012), which in turn affects people's compliance for preventive measures. Transparent risk communication is therefore of central importance and should be the goal of public communication.

Line 129 "*Earlier findings have indicated ~~that~~ perceived risks and different types of motivation as important predictors of compliance*"

Line 188 "*This period has been referred to as the "second wave" of infection spread and a number of infection control measures were in place at the time (Ministry of Health Services, 2020). The rise in COVID-19 cases and the appropriateness of the infection control measures was prominent in the public debate during the data collection period.*"

It would be helpful to readers to include details on average COVID-19 cases and on the infection control measures in the data collection period.

Discussion

Line 315 "*Thus, policy efforts to prevent infection spread may focus on increasing either self-interested or prosocial motivation to comply*".

Or both, to address the different information needs and preferences as well as motivations of the target groups.

Literature

Blastland M, Freeman AL, van der Linden S, Marteau TM, Spiegelhalter D (2020). Five rules for evidence communication. *Nature*. 2020;587(7834):362-4

Büchter RB, Fechtelpeter D, Knelangen M, Ehrlich M, & Waltering A (2014). Words or numbers? Communicating risk of adverse effects in written consumer health information: a systematic review and meta-analysis. *BMC medical informatics and decision making*, 14(1), 1-11.

Cokely ET, Feltz A, Ghazal S, Allan JN, Petrova D, & Garcia-Retamero R (2018). Decision making skill: From intelligence to numeracy and expertise. *Cambridge handbook of expertise and expert performance*, 476-505.

Kachurka R, Krawczyk MW, & Rachubik J (2021). Persuasive messages will not raise COVID-19 vaccine acceptance. Evidence from a nation-wide online experiment (No. 2021-07).

McDowell M, & Kause A (2021). Communicating Uncertainties About the Effects of Medical Interventions Using Different Display Formats. *Risk analysis : an official publication of the Society for Risk Analysis*, 41(12), 2220–2239. https://doi.org/10.1111/risa.13739

Volpp KG, Loewenstein G, & Buttenheim AM (2021). Behaviorally informed strategies for a national COVID-19 vaccine promotion program. *Jama*, 325(2), 125-126)

Wegwarth O, Schwartz LM, Woloshin S, Gaissmaier W, Gigerenzer G (2012). Do physicians understand cancer screening statistics? A national survey of primary care physicians in the United States. *Ann Intern Med*; 156(5): 340-349.

Zipkin DA, Umscheid CA, Keating NL, Allen E, Aung K, Beyth R, ... & Feldstein DA (2014). Evidence-based risk communication: a systematic review. *Ann Intern Med*, 161(4), 270-280.
