# Supplementary material for: Manipulating risk of infection and appeal to public benefit increase compliance with infection control measures in a hypothetical pandemic scenario
Source: PLoS One. 2022 Nov 22;17(11):e0274024. doi: 10.1371/journal.pone.0274024 (PMC9681092; doi:10.1371/journal.pone.0274024)
Supplement: Attachment — Submitted filename: Response to Reviewers.docx [file pone.0274024.s003.docx]

Dear editor,

We are very grateful to the editor and the two anonymous reviewers for taking time to consider our manuscript.

The reviewers made a number of comments and suggestions, all of which we found to be well-considered and meaningful. We have thoroughly revised all sections of the manuscript based on this review. How we have responded to each of the comments are detailed below, citing the revised manuscript text and their respective line numbers. Note that line numbers refer to the version of the manuscript where changes are not marked.

We hope that the editor and reviewers agree that these revisions have improved the clarity and the contribution of the manuscript.

Best wishes on behalf of both authors,

*Sebastian Bjørkheim*

# Editor

### Main issues:

### **1: Because the whole paper is about developing behavioral change measures (threatening and persuasion instead of information and communication), it should be written accordingly.**

*Reply: In our view, the main aim of the manuscript was to test framing effects of risk and motivation on compliance to infection control measures. Although the study may provide some insight that can be of value for public health communication, this was less of a focus in the previous version of the manuscript. However, this comment from the editor and other comments from both reviewers makes it clear that the manuscript could benefit from a stronger focus on it’s potential implication for communication strategies. We have taken this into consideration when revising the manuscript by editing the aim and implications of this study in the abstract, introduction, current study and discussion of results. This issue is also related to several of the other issues raised by the reviewers below.*

*Based on this comment, we have changed the last sentence in the abstract to emphasize that clarifies the focus on motives. It now reads (line 2-30): “These findings can inform health authorities about the motives underlying compliance with infection control measures during an infectious disease outbreak.”*

*In the Introduction section: To focus more on the underlying motives driving adherence to infection control measures, and deemphasized the public communication aspect, we have edited the “Perceived risk” section by adding information of how perceived risk, and risk literacy impact attitudes towards protective measures. In the “motivation” section, we have added some downsides to persuasive communication strategies. We have also made minor changes to direct emphasis away from health communication aspects towards the underlying motives that drive behavior.*

*In the “current study” section: We have made minor changes to emphasize that the aim is to test framing effects of risk and motivation on compliance. The section now reads (line 144-147) “Earlier findings have indicated that perceived risk, and the desire to protect oneself and others may be important predictors of compliance to infection control measures. The aim of the current study is to test causal relationships between risk framing and type of motivation on compliance with infection control measures, and to examine if these factors interact.

In the discussion section: We have edited the focus away from policy measures towards underlying motives impacting compliance in the first paragraph.*

### **2: There is the question whether other factors besides education, age, gender that have an influence on compliance with protection measures (e.g., SES, race/ethnicity) have been considered. Can they be identified using the Norwegian Citizen Panel?**

*Reply:*

*We agree that it could be valuable to also report the effect that other demographic factors may have had on compliance. We have added a section where we report the average score on the compliance to infection control measure in different demographic groups in Table 2.*

*In terms of SES, we have reported on how education level (measured as highest level of education completed) impacted compliance (See Table 2). Further, the dataset allows us to separate the sample into eight groups based on their (self-reported) gross annual income. We transformed this variable from eight groups into four groups to simplify the reporting. Please review the analysis script published on Osf.io to see the output from all eight groups. We have added descriptive statistics of compliance by income group in Table 2. Note that the gross annual income was converted from Norwegian Krone (NOK) to USD at a rate of 1 USD = 9.75 NOK (Date: 04.08.2022). This is reported in the table legend.*

*In terms of ethnicity, the panel population mainly consist of Norwegian citizens and only 5% (i.e. N < 200) report having a foreign citizenship or having parents from an immigrant background. Further, the majority of these are immigrants from other European countries. We thus have too few participants to investigate groups by different ethnicity and have not included this factor in Table 2. We are therefore unable to contribute to the very interesting and challenging issue of how ethnicity may influence compliance. In case other readers are also curious to the same issue, we have added the following sentence on (line 249): “There are too few participants with non-European ethnicity to investigate whether compliance vary by ethnic background.”*

### 3: What is missing from the limitations is that the results are not transferable to other countries, as trust in national public health authorities and past risk communication (especially the COVID-19 risk communication) influence the trust of the population and thus have an impact on compliance with future infection control measures.

*Reply:*

*We agree with the comment that this is a considerable limitation to the findings of this study. We have added the following to the limitations (line 421-425): “It has been reported that trust in government institutions as well as intra-individual trust is unusually high in the Scandinavian countries, and that this may impact compliance [39,40]. It could also be that the population’s experience with the public health communication in past outbreaks has an impact on compliance with current infection control measures. Such factors should be taken into consideration when generalizing the results to other settings.”*

*Note that we have previously published an article (in Norwegian) on the issue of trust in authorities during the COVID-19 pandemic using data from the Norwegian Citizen panel (*<https://doi.org/10.18261/issn.2464-3076-2021-02-06>)*.*

### 4: Further, there is no discussion in the limitations section on how the under-representation of certain groups (e.g., with low education) can be reduced in the future.

*Reply: Thank you pointing this out. We agree that this could be mentioned in the manuscript.*

*Our sample comes from a nationally representative panel that has been running for several years to answer various social science questions. It is outside of our project’s scope to adjust the composition of the panel. The relatively modest deviation from perfect representation is clearly reported in the manuscript. The open dataset that is made available for the reader is provided with weighting variables for age, education level and gender in each data collection round. This allows the interested reader to re-run the analyses while adjusting for the imperfections in representation. For a more direct approach, the descriptive results section for compliance by different demographic groups can be consulted to see what impact various adjustments in representation would have for the overall compliance. We have added that the dataset is provided with weighting variables on line 185: “*The dataset is provided with weighting variables for demographic deviations from representativity.*”*

*Furthermore, we feel that this issue is somewhat besides the research question that this paper is intended to address. The research aim of the current paper was to test framing effects of risk and motivation on compliance. It was thus not an aim to report the level of self-reported compliance among a completely accurately represented sample of the Norwegian adult population. Although a large and mostly representative sample adds validity to answering this question, we can still address our intended research question despite the modest deviations from perfect representativeness. Nevertheless, we have added the following under Implications and Future research: “The panel would benefit from a targeted recruitment of people with lower levels of education and younger age to improve representativity for future studies.”*

Reviewers' comments:

## Reviewer's Responses to Questions

**Comments to the Author**

1. Is the manuscript technically sound, and do the data support the conclusions?

The manuscript must describe a technically sound piece of scientific research with data that supports the conclusions. Experiments must have been conducted rigorously, with appropriate controls, replication, and sample sizes. The conclusions must be drawn appropriately based on the data presented.

Reviewer #1: Yes

Reviewer #2: Partly

2. Has the statistical analysis been performed appropriately and rigorously?

Reviewer #1: Yes

Reviewer #2: Yes

3. Have the authors made all data underlying the findings in their manuscript fully available?

The [PLOS Data policy](http://www.plosone.org/static/policies.action#sharing) requires authors to make all data underlying the findings described in their manuscript fully available without restriction, with rare exception (please refer to the Data Availability Statement in the manuscript PDF file). The data should be provided as part of the manuscript or its supporting information, or deposited to a public repository. For example, in addition to summary statistics, the data points behind means, medians and variance measures should be available. If there are restrictions on publicly sharing data—e.g. participant privacy or use of data from a third party—those must be specified.

Reviewer #1: Yes

Reviewer #2: Yes

4. Is the manuscript presented in an intelligible fashion and written in standard English?

PLOS ONE does not copyedit accepted manuscripts, so the language in submitted articles must be clear, correct, and unambiguous. Any typographical or grammatical errors should be corrected at revision, so please note any specific errors here.

Reviewer #1: Yes

Reviewer #2: Yes

5. Review Comments to the Author

Please use the space provided to explain your answers to the questions above. You may also include additional comments for the author, including concerns about dual publication, research ethics, or publication ethics. (Please upload your review as an attachment if it exceeds 20,000 characters)

# Reviewer #1:

## General issues:

### 1: Thank you for the opportunity to review the manuscript. The work is very interesting in terms of exploring different motivations for adherence to infection control measures during pandemics. However, it is unclear what recommendations for policy makers can be derived from the study results and to what extent best practices and ethical standards of effective (evidence-based) risk communication have been considered, aiming at empowering citizens in democratic societies to make informed decisions. For instance, evidence suggests that persuasive strategies threaten trustworthiness and credibility of the communicator and of vaccinations (e.g., Blastland et al. 2020; Kachurka et al. 2021; Volpp et al. 2021. Therefore, trust in public health authorities is a key factor in behaviour and compliance. We suggest, that different strategies of communication with the public as well as their chances of success and consequences should at least be discussed.

*Reply:*

*As the reviewer points out, the manuscript’s main objective is to explore the role of risk and prosocial motivation on compliance, and not to advance the field of public health communication or to directly offer policy recommendations. Hence, we agree that it was unclear in the previous version of the manuscript what recommendations policy makers should derive from this study. We see that the study may be of value in assisting health authorities to learn more about which factors influence why people comply with public health advice. We agree that a discussion of evidence-based risk communication would improve the manuscript. We have therefore tried to be clearer on this issue throughout the manuscript. See also our response to the editor’s first comment.*

*We also think the reviewer’s issue about potential downsides of persuasive communication strategies is well made. We have implemented this issue by adding to the introduction on line (100-102) “However tailoring health communication to persuade people into adopting health protective behavior may also have downsides, such as reducing the credibility of the recommendation and reducing trust in the messenger [28]. Moreover, emphasizing arguments in favour of taking precautions has also been found to be ineffective in increasing vaccine uptake [29].”*

*We agree that the role of trust in public health authorities should be discussed in the manuscript and that high trust may limit the interpretation and limit the generalizability of the results. We address this issue in our response to issue number 2 below.*

### 2: What is missing from the limitations is that the results are not transferable to other countries, as trust in national public health authorities and past risk communication (especially the COVID-19 risk communication) influence the trust of the population and thus have an impact on compliance with future infection control measures. Further, there is no discussion in the limitations section on how the under-representation of certain groups (e.g., with low education) can be reduced in the future. There is also the question whether other factors besides education, age, gender that have an influence on compliance with protection measures (e.g., SES, race/ethnicity) have been considered. Can they be identified using the Norwegian Citizen Panel?

Reply:
*We agree that trust may play an important role in the public’s adherence to infection control advice from health authorities, and that there may be important variation in trust between regional settings. To take this into account, we have added the following to the limitations section (line 421-425): “It has been reported that trust in government institutions as well as intra-individual trust is unusually high in the Scandinavian countries, and that this may impact compliance [39,40]. It could also be that the population’s experience with the public health communication in past outbreaks has an impact on compliance with current infection control measures. Such factors should be taken into consideration when generalizing the results to other settings.”*

*Our sample comes from a nationally representative panel that has been running for several years to answer various social science questions. It is outside of our project’s scope to adjust the composition of the panel and the relatively modest deviation from full representativity is clearly reported in the manuscript. The open dataset that is made available for the reader is provided with weighting variables for age, education level and gender in each data collection round. This allows the interested reader to re-run the analyses while adjusting for the imperfections in representation. For a more direct approach, the new Table 2 can be consulted to see what impact various adjustments in representation would have for the overall compliance.*

*See also our response to the editor’s second comment on the same issue.*

*We have added the following under Implications and Future research: “The panel would benefit from targeted recruitment of people with lower levels of education and younger age to improve representativity for future studies.”*

## Specific issues:

Please find below a few comments on some of the sections and phrases.

### Specific issue 1: Abstract In general, the methodology could be explained a bit more: sample, design, analyses. It is not clear from the abstract what the main objective is and in how far the results of this study can inform future communication of health authorities. In 1.3.1 you wrote “The aim of the current study is to test causal relationships between risk of infection and type of motivation on complying with infection control measures, and to examine if these factors interact.” and in line 119 “Knowledge about the relative importance of self-interested and prosocial motives in different risk scenarios can inform health authorities about communication strategies during different phases of disease outbreak.” Maybe you can add this to your abstract.

*Reply:*

*We agree that the aims may have been unclearly stated in the previous version of the manuscript. The main objective of this study is to test the framing of risk and prosocial motivation on compliance to infection control measures, and not to provide policy recommendations for health authorities. Nevertheless, the results may provide some insight that can be of value in that field. Comments from both reviewers and editor makes it clear that we should further emphasize this aspect of the paper. We have taken this into consideration when revising the manuscript by editing aim and implications of this study in the abstract, introduction, aim and discussion of results. Please also see our response to the editors first comment.*

*To clarify the aim and its implications, we have changed the sentence you are referring to on line (134, previously 119). It now reads: “Knowledge about the relative importance of self-interested and prosocial motives in different risk scenarios can inform health authorities about how people respond to infection control measures during different phases of disease outbreak”*

*Hopefully this makes it clearer that we are offering up evidence for how people respond to risk and motivation framing, which may be of value for health authorities. Similarly, we have also changed the last sentence of the abstract to clarify this. “These findings can inform health authorities about the motives of compliance to infection control measures during an infectious disease outbreak.».*

*As for the general comment about making the methodology clearer in the abstract, we have highlighted the study design by adding “…with a 2-by-2 between subject design…” to the abstract. To highlight the sample we have added “from a large representative Norwegian sample (N = 2533)“ to the abstract*.

### Specific issue 2: Line 17 „Perceiving the situation as threatening and seeing public benefits to complying may increase the public’s motivation to comply.“ It is not clear what the conclusion for political actions could be? Should it be the aim of political communication be to raise fear (even if there is no reason for it or when there is a lot of uncertainty)? We view this statement critically in the light of evidence-based risk communication, which aims at transparent and balanced risk communication to enable informed decisions. If persuasion is the recommendation, it should be made more clear in the abstract.

*Reply: As discussed above, we agree that the aim of the study could have been more clearly stated. We have tried to improve this by editing the manuscript in the abstract, discussion, limitations section. The study did not aim to provide specific recommendations to health authorities and be used to guide health communication as such, but to provide insights into how people’s motivation may change depending on how health information is presented (framing effect of risk and motivation). To make this clearer, we have edited the line you single out above. It now reads: “Perceiving the situation as threatening and seeing public benefits to complying may be underlying motivations for compliance.”*

### **Specific issue 3: Introduction, Line 38 “Although the probability of an event and the expected outcome can be established statistically, perceived risk has been shown to deviate from objective measures of risk (2,3). How threatening a pandemic is perceived to be, may be influenced by how public authorities communicate the risk scenario.”**

**The statistical probabilities of occurrence cannot be** determined for all events, as also described in the paper by Mousavi & Gigerenzer 2014 that you cite. Especially during the COVID-19 pandemic, a great deal of uncertainty existed at the beginning (and to some extent still exists). Therefore, a key factor influencing perceived risk is how risks and uncertainties are communicated by public authorities. However, evidence-based and user-centered risk communication not only includes transparent and balanced communication of probabilities, but also indicates the uncertainties, and takes into account the needs of different target groups). People better understand risks when, for instance, risk probabilities are communicated numerically instead of verbally (for example, “5 in 100 people will be infected” can be grasped more concretely than “The risk of infection is low or high”), since people tend to interpret verbal probability statements differently. Simple frequencies or per- centages (for example, 5 in 100 or 5%) are more comprehensible than probabilities or 1-in-x formats (for in- stance, 1 in 20). By communicating both numerator and denominator, it can be conveyed whether the risk is big or small. Moreover, when comparing risks, the denominator should be kept the same (for example Is 100). Relative risk reductions are unclear (“The intervention reduced the number of infections by 20%”), in- stead, absolute risk reductions are recommended (”The intervention reduced the number of infections from 5 in 100 people without the treatment to 4 in 100 people with the treatment”) (e.g., Zipkin et al. 2014, Büchter et al. 2014). The latter illustrate the absolute size of a risk. If there is insufficient information to report numbers, the reason for this should be stated. Visual presentations can be a beneficial supplement or substitute for numerical or verbal information on risks. They can facilitate comprehension, especially for people with low numeracy or reading skills (e.g., McDowell et al. 2021).

*Reply:*

*Thank you for pointing this out, and we agree that the literature on perceived risk could be improved by discussing evidence-based risk communication. We have now altered the manuscript to focus more on the motives of people responding in a risk scenario, and based on the reviewer’s comment we have adjusted the introduction of perceived risk. This section now reads “[1]. Although the probability of an event and the expected outcome can be established statistically in some cases, perceived risk has been shown to deviate from objective measures of risk [2,3].The individual perception of risk may depend on statistical literacy and the way in which risk is communicated[4]. Information about personal risk may thus be overestimated or underestimated as a consequence of how public authorities communicate a risk scenario. While the optimal method for conveying health risks has yet to be agreed upon, it has been found that visual aids and information about absolute event rates improves risk understanding among patients [5]. As mentioned above, since the stated aim of the current study was to test the effect of risk framing and motivational emphasis on compliance, we do not feel that a further discussion of the details of different risk communication strategies is justified.*

### Specific issue 4: Line 60 “If feeling threatened is essential for compliance, this would justify that health authorities emphasize personal and societal risks when promoting infection control measures.“ In the light of evidence-based risk communication, the benefits and harms of both perspectives (personal and societal risks) should be communicated if this is in the interest of the addressees.

*Reply:*

*Thank you for pointing this out. We agree that the phrasing was unclear, and we have edited the line the reviewer is referring to. It now reads “If feeling threatened is essential for compliance, emphasizing personal and societal risks when promoting infection control measures may increase compliance”

Note that we have changed the central focus of the manuscript to emphasize underlying motives and to a lesser degree infringe on health communication (as discussed above).*

### Specific issue 5: Line 112 “How people perceive risk is related to their ability to control their level of exposure and it may thus be difficult to determine the impact of perceived risk on compliance from correlational data alone (25).” How people perceive risks also depends on their risk literacy, which in turn is influenced, for example, by their statistical skills and the way in which risks, and uncertainties are communicated (e.g., Cokely et al. 2018). If risks are not correctly understood, risks can be overestimated or underestimated (e.g., Wegwarth et al. 2012), which in turn affects pople's compliance for preventive measures. Transparent risk communication is therefore of central importance and should be the goal of public communication.

*Reply: We agree that risk literacy also play an important role in how people perceive risk scenarios and that this should be noted in the manuscript among the factors that influence risk perception. Therefore we have added the following sentence in the “Perceived risk” section: “An individual’s perception of risk depends on their statistical literacy and the way in which risk is communicated(4). Information about personal risk may thus be overestimated or underestimated depending on how public authorities communicate the risk scenario”.*

### Specific issue 6: Line 129 “Earlier findings have indicated that perceived risks and different types of motivation as important predictors of compliance”

*Reply: We have revised this sentence. It now reads: “Earlier findings have indicated that perceived risk, protecting oneself and protecting others may be important predictors of compliance to infection control measures»*

### Specific issue 7: Line 188 “This period has been referred to as the “second wave” of infection spread and a number of infection control measures were in place at the time (Ministry of Health Services, 2020). The rise in COVID-19 cases and the appropriateness of the infection control measures was prominent in the public debate during the data collection period.” It would be helpful to readers to include details on average COVID-19 cases and on the infection control measures in the data collection period.

*Reply: We agree that it would be helpful to know which infection control measures were instituted at the time of data collection. While listing all of them in detail may be excessive, we have summarized them in general terms: «At the time of the data collection, the Norwegian health authorities advised the public to practice social distancing, to avoid domestic and international travel, comply with quarantine and isolation requirements and take hygiene precautions. In addition, the government restricted organized sport and other recreational activities. We have also included a sentence about the increase in number of cases that we are referring to as the “second wave”. The sentence now reads: “.”*

### Specific issue 8: Discussion: Line 315 “Thus, policy efforts to prevent infection spread may focus on increasing either self-interested or prosocial motivation to comply“. Or both, to address the different information needs and preferences as well as motivations of the target groups.

*Reply: We agree that this could be a worthy topic of discussion, but as we have discussed above, the original aim of the manuscript was to highlight the effect of risk scenario and prosocial framing, rather than to focus on health communication strategies. As mentioned above, we have made some changes to further emphasize possible policy recommendations of understanding the underlying motives people have for complying.*

*Nevertheless, the reviewer brings up a good point about people can be motivated by self-interest, by prosocial motivations, or a combination thereof. Policy makers should take this into consideration when designing risk communication, in order to address the information needs and preferences as well as motivations of the target groups.*

*Also, we have altered the sentence in question, to “*Thus, compliance may vary depending on whether a message emphasizes self-interested or prosocial reasons, or both, for compliance.

# Reviewer #2:

## The authors submitted a scenario-based experimental study that varied infection risks. They renew a traditional finding: that perceived risk from a disease is associated with increased compliance to preventive measures. I am suggesting to resubmit a modified manuscript.

## Major issues:

### 1: The manuscript in the current form does not convey a strong contribution. Compared to existing evidence: The distinct association of risk perception (probability not always, severity yes) with reported preventive measures was shown before (e.g. COSMO studies in Germany and Spain; e.g. Rattay et al.). Now doing a study on risk-dependent intentions to comply (weaker endpoint) does not add much.

Reply:

*We understand that the contribution of the current manuscript should be seen in reference to the wealth of other studies examining the relationship between perceived risk and compliance. We have added the studies the reviewer mentions, in addition the discussion of similar studies in the “Perceived risk” section of the Introduction.*

*We further agree that there are disadvantages to using self-reported intention to comply as an outcome measure. This is briefly discussed in the “Limitations” section: “The experiment is limited in that the outcome variable measured intention to comply with infection control measures rather than actual behavior outcomes. Stated intentions may be influenced by social desirability bias such that participants respond in a manner that is viewed as favorable by others (36).“*

*While the COSMO study that the reviewer refers to appears to have as its main research question how education level may influence compliance, this was not one of the main research questions for our study. The design is also notably different in that we use a survey-experiment and not correlational data to determine the relationship between perceived risk and compliance. It should also be noted that we conduct our study on a representative Norwegian sample that may impact the results (see below). However, we have added the Rattay et al. (2021) article to the literature section on perceived risk.*

*On the other hand, we do think that the current study makes valuable contributions to the literature on this issue. Given that (as the reviewer points out) there already is a literature suggesting predictors for compliance, we think the time is ripe for making confirmatory tests of preregistered designs and hypotheses. In our opinion, preregistered studies may make stronger claims about the direction and magnitude of a relationship than studies that do not explicitly state their analysis plan before data collection. As far as we can see, the articles mentioned by the reviewer do not mention any preregistration for the hypotheses mentioned in the COSMO article, and it may therefore be difficult to discern the confirmatory statements from exploratory findings (although we did find a* [*preregistration*](https://www.psycharchives.org/en/item/e5acdc65-77e9-4fd4-9cd2-bf6aa2dd5eba) *by the COSMO data collection service with general statements about some of the relationships they intended to explore). We would argue that a contribution of the current study is the preregistered testing of hypotheses derived from the established literature.*

### 2: Intention to comply with public health measures in the light of societal benefits, while that directly depends on the perceived-benefit harm ratio of public health measures, does also add not much. Generally, the manuscript has to be settled more in the existing COVID literature or at least protection-motivation literature.

*Reply: We agree that we should have provided a more detailed review of the existing literature on health-related motivated behaviour and how it relates to our current study. We have expanded parts of the introduction to include literature on evidence-based risk communication and downsides of persuasive health messaging. See our response to issue number one raised by the editor and issue number one raised by reviewer number one.*

*We have also expanded the literature section on perceived risk to emphasize that an association between perceived risk of COVID-19 and compliance to infection control advice has been documented in several countries. For instance, see line 66 “The association between perceived risk and knowledge of COVID-19, and the adoption of protective measures has been found in a number of countries [e.g. in China; 15; in Saudi Arabia; 17; Egypt and Nigeria; 18; and in Germany; 19].*

*However, as we pointed out in our response to the first issue raised by the editor, we see the main aim of the manuscript to focus more on the underlying motives for compliance. See more on this issue in our response to this reviewer’s third major issue below.*

### 2: Meaningful results: Once you work with thousands of participants from quasi-representative surveys, for main effects significance testing is secondary to meaningful effects. In 3.2.1.: Main effect for high risk on compliance intention: partial eta square .03. What is the interpretation of not finding a larger effect? Extreme example 3.2.2.: with 2,521 degrees of freedom, can we speak about an effect with partial eta square <.01? This is particularly important in the light of the interaction of motivation and risk: ones we acknowledge the relationship (higher risk --> higher personal risk perception --> more compliance with measures that protect me against this risk) -- why this was not shown [please adjust the y-axis range of Figure 1]? I do not have the answer. But this could be the key finding (e.g. confirming that probabilities are less important than severity in this context?). You should make use of the large dataset and explore potential confounders for that. Related to that - where are the descriptive statistics of risk perception and prosociality (manipulation checks)? The second paragraphs about small effects in limitations and in implications cannot replace that.

*Reply: We think the reviewer raises an important issue here, in pointing out that effect sizes may be more important than significant results, in particular when working with large samples such as here. We further agree that the small effect sizes seen in the current study warrants some further discussion and caveats. We have tried to make this clearer in the limitations section of the revised manuscript and we have also noted the small effect sizes in the abstract. For example, we have emphasized this point in the limitations section by adding on line 418 “Thus, the small effect sizes in our results may indicate that the practical impact of the communication framing on the level of compliance to infection control measures is limited.”*

*If we understand the comment correctly, the small magnitude of the effect sizes is related to the first major issue raised above (i.e., whether the study convey a strong contribution). Although we agree that this should have been discussed in more detail, it is also worth noting that preregistered studies tend to find smaller effect sizes than what has previously been shown in the literature (*[*Camerer et al., 2018*](https://www.nature.com/articles/s41562-018-0399-z)*). If the “true” effects of these manipulations are smaller than what has been shown in the past, the present study contribute to the literature in this regard.*

*The paragraph in the limitations section about small effects that the reviewer mentions was not meant to replace the lack of practically meaningful effect sizes. Rather, our intention there was to highlight that little of the variance is explained by the experimental manipulations, and that variation in compliance may therefore be explained by other factors. We have edited this paragraph somewhat to make it clearer how our intentions were aligned with the issue the reviewer raises. However, it could also be noted that on a population level, even small changes in people’s intention to comply with infection control measures may lead to a considerable difference in overall infection rates in a population. If reliable, such effect could thus be of clinical significance, although the effects sizes are small. We have added a brief mention of this argument on line 438. It now read “*Although the effect sizes were small, the sample size and approach indicate robust results that to a population level may nevertheless translate to increased compliance for many people, and consequent quality-of-life improvements for those who avoid infection.”*.*

*The reviewer also mentions that it would be reasonable to expect an interaction of risk and motivation. We completely agree, and this was in fact one of our preregistered expectations for the results (H3). Given the surprising null-finding for this interaction, we agree that it warrants more of a discussion in the manuscript than what was previously there. We have edited the “Discussion” and “Limitations” sections to emphasize that this unexpected finding. We further agree with the reviewer that it may be explained by the fact that we manipulated the probability side of the risk term and not the consequence side. We have suggested that this possible explanation should be explored in future studies on line 390-397: “Our choice to focus on probability rather than (or addition to) a focus on consequences may be defensible as the consequences of being infected may be somewhat similar (and somewhat uncertain) across our participants. Further, any differences (e.g., due to believing one is part of an at-risk group) should be randomized across our experiment groups. However, the focus on the probability side of risk may be part of the reason why we did not observe an interaction effect between risk and motivation. Future studies could manipulate the consequence instead of or in addition to the probability of contracting the disease to investigate if this aspect of perceived risk has different main effect or interaction with motivational framing.”.*

*As for the Y-axis of Figure 1: We agree with the general rule-of-thumb that an axis should show the entire range of the scale. However, in our data approximately 84% of the sample responded either “Completely certain” or “Quite certain” (the two uppermost response options) while only 3% selected one of the four options suggesting less compliance. This creates a ceiling effect, which may have made the effects difficult to discern, and may have contributed to providing the very small effect sizes as the reviewer mentions above. While this may have been unfortunate for the experiment, the very high intention to comply with infection control measures is in line with what has been found previously in the COVID-19 pandemic in Norway. We have discussed this in a previous publication (Sætrevik, 2021). To visually present the differences between the conditions it is a necessity to adjust the Y-axis. The fact that the y-axis is truncated is clearly reported in the figure legend. Also, in the hypothesis testing part of the manuscript, we are mainly interested in detecting the difference between the conditions, and not to report the full distribution of responses. As we see it, Table 1 and Figure 1 provide complementary information for the reader in this respect.*

*We have added the descriptive statistics for perceived risk of COVID-19 infection and prosocial motivation for complying with the infection control measures for the explorative analyses. “The answers were reported on a five-point scale ranging from “very low” to “very high”, and overall the participants considered it moderately likely that they would be infected (M = 2.52, SD = .92)”, and “Overall, the participants reported a very high degree of motivation to protect others (M = 4.52, SD = .63)”.*

### 3: Conclusion, starting from the „starting point“: "the public must be informed about the infection risk and be motivated to comply with infection control measures". It lacks the main point: "the public must be informed about the benefits and harms of complying (and not complying) with infection control measures". As long as this is not clarified, "these findings can[not] inform communication strategies".

*Reply: We agree that the perceived benefit and cost of complying needs to be added and discussed in the manuscript. It is indeed one of the main aims of the paper to show the value of not only give the public information about a pandemic and the infection control measures, but to motivate them for action by pointing out the benefits and harms of acting on the information (i.e., manipulating personally experienced risk and consequences). In editing the manuscript, we have to a larger extent emphasized the underlying motives of compliance and downplayed the health communication aspects. We have added perceived “cost” to the factors mentioned in the introduction. Please see our response to the editors first comment.*

### If the whole paper is about developing behavioral change measures (threatening and persuasion instead of information and communication), it should be written accordingly.

*Reply: Please see our response to the editor’s first comment on the same issue.*

### Related to that, goal of communication is to adjust risk perception of recipients in line with the objective risk (so, accurate risk perception), not to increase disease risk perception beyond, which can cause harm, similar to fear induction.

*Reply: We agree that the goal of the authorities’ health communication in most cases should be to align the public’s understanding of risk with what is the expert opinion about what an optimal level of risk should be (and that there may be downsides in overly high perceived risk). We have included a brief mention of this general issue in the Introduction. It reads: “[1]. Although the probability of an event and the expected outcome can be established statistically in some cases, perceived risk has been shown to deviate from objective measures of risk [2,3]. The individual perception of risk may depend on statistical literacy and the way in which risk is communicated[4]. Information about personal risk may thus be overestimated or underestimated as a consequence of how public authorities communicate a risk scenario. While the optimal method for conveying health risks has yet to be agreed upon, it has been found that visual aids and information about absolute event rates improves risk understanding among patients [5].*

*More specifically, we have highlighted the issue of the downsides of persuasive communication strategies in the “motivation” section (line 100-102): “However tailoring health communication to persuade people into adopting health protective behavior may also have downsides, such as reducing the credibility of the recommendation and reducing trust in the messenger [28]. Moreover, emphasizing arguments in favour of taking precautions has also been found to be ineffective in increasing vaccine uptake [29].”*

*In the current study, our aim was to map out two different hypothetical risk scenarios (one high risk and one low risk) and detect if motivational messaging influenced compliance in the two scenarios. This may provide insight into how people respond to the framing of infection risk, but does not advocate for misleading communication strategies. We thus do not make any assumptions of what “the optimal” level of perceived risk should be, but rather try to learn more about the factors that influence it. We have tried to make this clearer throughout the manuscript. For more on this, see our response to the first issue raised by the editor.*

## Minor issues:

### - "Manipulating" seems unnecessary as the title expresses the finding

*Reply: For now, we would prefer to retain “Manipulating” as it helps to distinguish this study from studies with correlational designs on the same relationships.*

### - Threatening and fear is not equal to risk perception - this needs to be clearly disentangled throughout the paper, e.g. p.3, l.40, when you talk in a paragraph about risk perception

*Reply: We agree that “threat” and “fear” should be differentiated from “risk perception”. We have edited the manuscript to substitute “threat” for “risk” or “perceived risk” in a number of places where the concepts may be interchanged. We do not refer to the emotion of fear anywhere in the manuscript as far as we can see. As for the line that the reviewer is referring to, we clarified how threat is used in reference to the framework of the protection motivation theory, and how this suits a pandemic risk scenario on line 52 “During a pandemic, threat severity and threat vulnerability may correspond to how people asses the likelihood of being infected and how severe such an event would be».*

### - p.6 l.129: check grammar of the sentence please

*Reply: As the sentence do not add anything beyond the links to the data, material, and preregistration, that we have referenced to in other places, we have decided to remove it.*

### - p.8 l.161-164: percentages of what (absolute, relative overrepresented)?

*Reply: Here we mean percentage deviation from a perfectly representative sample of the Norwegian population. The percentages are compared to population statistics. Please consult the documentation of the data collection procedure that is linked to on line 176. We have edited the first line to make this clearer. It now reads “Compared to a perfectly representative sample of the Norwegian population, there is some underrepresentation in terms of age, education level, and geographical residency.”*

### - Hypotheses and Design: what is "level of infection risk"? The stimuli could have produced a mixed understanding of perceived probability of infection (exposure risk in the low risk condition - "only a few people") and perceived severity of infecting (high risk: "serious outbreak of infection"). For another study, „many people“, „most of the people“, „nearly everybody“ would be better comparable. Was there information for the participants, explaining why following infection control advice stops the outbreak?

*We agree that the “high risk” condition could have been more clearly explained. This may partly have been due to insufficient translation of the original Norwegian survey materials as outbreak and (Norwegian: “alvorlig utbrudd”) do not translate perfectly. The high-risk framing does refer to many people being infected, and not the severity of the infection. It is also possible that the alternative design that the reviewer suggests of more directly comparing the number of people infected in both conditions could have offered different results.*

*However, both the risk scenarios used in the current study do emphasize that the infection risk concern people similar to the respondent in the latter part of the description (i.e. in the area where you live among those in the same life situation as you).* *The risk variable has two levels in the experiment. “Level of infection risk” indicate the two risk scenarios (one that emphasize that relatively few people are infected with a contagious disease, and one where there is a serious outbreak).*

*As for the last question in the comment, we did not present to the participants a mechanism by which following the advice would stop the outbreak. Instead, the potential benefits of following the advice was varied between the conditions. For the personal benefits condition, we described that you will be safer, and for the prosocial benefits condition, we described that following the advice would stop the outbreak. This is now stated more clearly on line 230-231: “This was framed as either personal benefits: “that you will be safer if you follow all infection control advice” or prosocial benefits: “it is important that you follow all infection control advice in order to stop the outbreak”.*

- p.10 l.210 and 211: check grammar of the stimulus sentences please

*Reply: We have made minor changes to these sentences.*

### - consider removing sentences and expression, "something is significant", when you report statistics anyway and as you would not phrase non-significant differences as differences without comment.

*Reply: Thank you for pointing this out. We have removed “significant” from the results section and instead presented our findings by referring to a “small effect” or “no effect” wherever appropriate.*

### - ethics: the study procedure did not deviate from the standard practice of the Norwegian Citizen Panel - with health data - complying in ongoing pandemic measures, experimental design? Could you attach the study consent form?

*Reply: A translated version (from Norwegian to English) of the consent form has been attached to the submission.*

### - what is the message of table 1 beyond the aggregate reporting? consider removing.

*As you point out the main contribution of the table is the aggregate reporting of the responses. First, the table underlines the distribution of responses that we refer to when we present the adjusted Y-axis in figure 1. Approximately 84% of the sample responded “Completely certain” or “Quite certain” that they would always comply with the measures, creating a ceiling effect. We are mainly interested in detecting the difference between the conditions, not to report the full distribution of responses in the hypothesis testing part of this paper. As this information is not stated in the main text and provides an immediate way to communicate the overall baseline for interpreting the rest of the results, we think it may be valuable to retain Table 1.*

### - 3.2 can be presumed by the reader, could be skipped

*We agree that this can be presumed by the reader after reading the “Results” section, but it may be helpful for some readers to get the findings summarized in simple language without reference to test statistics. For now, we would prefer to retain this section.*

### - p.17, l.382 "overstates"?

*Reply: Framing effects that use intentions as the outcome variable tend to exaggerate the subsequent change in behavior. We have replaced “overstate” with “exaggerate” in line 416.*

- some literature:
Rattay, P., Michalski, N., Domanska, O. M., Kaltwasser, A., De Bock, F., Wieler, L. H., & Jordan, S. (2021). Differences in risk perception, knowledge and protective behaviour regarding COVID-19 by education level among women and men in Germany. Results from the COVID-19 Snapshot Monitoring (COSMO) study. Plos one, 16(5), e0251694.
Betsch, C., Wieler, L., Bosnjak, M., Ramharter, M., Stollorz, V., Omer, S., ... & Schmid, P. (2020). Germany COVID-19 Snapshot MOnitoring (COSMO Germany): Monitoring knowledge, risk perceptions, preventive behaviours, and public trust in the current coronavirus outbreak in Germany. <https://www.psycharchives.org/en/item/e5acdc65-77e9-4fd4-9cd2-bf6aa2dd5eba>

Beca‐Martínez, M. T., Romay‐Barja, M., Falcón‐Romero, M., Rodríguez‐Blázquez, C., Benito‐Llanes, A., & Forjaz, M. J. (2021). Compliance with the main preventive measures of COVID‐19 in Spain: The role of knowledge, attitudes, practices, and risk perception. Transboundary and Emerging Diseases.

Betsch, C., Korn, L., Burgard, T., Gaissmaier, W., Felgendreff, L., Eitze, S., ... & Bosnjak, M. (2021). The four weeks before lockdown during the COVID-19 pandemic in Germany: a weekly serial cross-sectional survey on risk perceptions, knowledge, public trust and behaviour, 3 to 25 March 2020. Eurosurveillance, 26(42), 2001900.

Felix Rebitschek
I always sign my reviews
